# Bilateral subcapital femoral fractures associated with transient osteoporosis of the hip in pregnancy: a case report

**DOI:** 10.1016/j.tcr.2026.101325

**Published:** 2026-04-21

**Authors:** Zoltan Cibula, Jozef Cabala, Milan Cipkala, Peter Lisý, Juraj Cabala

**Affiliations:** a Jessenius Faculty of Medicine in Martin, Comenius University in Bratislava, Martin, Slovak Republic; b University Department of Orthopaedic Surgery, University Hospital Martin, Kollarova 2, Martin, 036 59, Slovak Republic

**Keywords:** Bilateral femoral neck fractures, Transient osteoporosis, Femoral neck system

## Abstract

Bilateral femoral neck fractures associated with transient osteoporosis of the hip (TOH) during pregnancy are rare. We report a case of a 46-year-old woman at 35 weeks of gestation who presented with progressive bilateral hip pain and impaired mobility without preceding trauma. Only a limited number of similar cases have been reported, underscoring the importance of considering TOH in the differential diagnosis of pelvic girdle pain in pregnant and postpartum women. Magnetic resonance imaging (MRI) remains the gold standard for diagnosis. Initial conservative management with analgesia and supportive care was unsuccessful, and the patient's condition deteriorated, resulting in immobility and severe pain. Delivery was performed via cesarean section based on a multidisciplinary team decision. Postpartum MRI revealed bilateral subcapital femoral neck fractures. Surgical treatment consisted of bilateral closed reduction and internal fixation using the Femoral Neck System (FNS), achieving satisfactory reduction and stable fixation. Six months postoperatively, clinical deterioration of the left hip required implantation of a non-cemented total hip arthroplasty due to partial avascular necrosis of the weight-bearing portion of the femoral head. Identified risk factors included advanced maternal age, obesity, pregnancy achieved through assisted reproductive technology (IVF), hypothyroidism, and presumed osteopenia. This case highlights the diagnostic and therapeutic challenges of TOH and emphasizes the importance of early recognition to prevent severe complications.

## Introduction

Transient osteoporosis of the hip during pregnancy (TOHP) represents a rare clinical entity whose incidence, etiology, pathogenesis, risk factors, and optimal treatment methods have not yet been clearly elucidated. In pregnancy, it most commonly manifests in the third trimester or in the immediate postpartum period. Early MRI diagnosis plays a crucial role in preventing serious associated morbidity. The most severe complications include subcapital femoral neck fractures and avascular necrosis of the femoral head.

This report describes a primigravida with transient osteoporosis of the hip complicated by bilateral subcapital femoral fractures. Several potential risk factors were identified, including advanced maternal age, obesity, pregnancy achieved by in vitro fertilization, concomitant hypothyroidism, and presumed osteopenia based on a history of low-energy fractures.

This case highlights a rare presentation of transient osteoporosis of the hip during pregnancy with bilateral subcapital femoral fractures. Treatment consisted of osteosynthesis using the Femoral Neck System (FNS), which, to authors knowledge, represents the first reported use of this technique in such a rare clinical case.

The patient was informed that data from the case would be submitted for publication and provided consent.

## Case report

### Case history

This case involves a 46-year-old primigravida of advanced maternal age (primigravida vetus) whose pregnancy was achieved through assisted reproductive technology by in vitro fertilization (IVF). The patient was admitted to the gynaecology department at 35 weeks of gestation due to gradual onset of a non-traumatic pelvic pain, predominantly affecting both groin and anterior thigh.

The pain had persisted for approximately one week, with initial onset in the right hip. At admission, the patient was ambulatory only with the assistance of two crutches. Clinical examination revealed bilateral hip pain, even during passive range of motion. Despite hip pain during movement, the range of motion was only minimally restricted. Bilateral perimalleolar oedema was present, while the lower extremities remained neurovascularly intact.

From a gynaecological perspective, the patient was asymptomatic, reporting normal fetal movements, with no vaginal bleeding and no uterine contractions.

The patient had a history of hypothyroidism, treated with levothyroxine (Euthyrox, Merck, Darmstadt, Germany) 12.5 μg once daily. Her history also included previous low-energy fractures of the distal radius and distal humerus.

Initial imaging studies, including plain radiography of the pelvis, excluded femoral neck fractures, and venous ultrasonography of the lower extremities revealed no pathological findings. Neurological examination ruled out radicular pathology. Laboratory investigations of bone metabolism, including parathyroid hormone levels, serum and urinary calcium, alkaline phosphatase, serum osteocalcin, and hydroxyproline, were all within reference ranges.

Despite conservative management with analgesics, non-steroidal anti-inflammatory drugs, and restriction of weight bearing activities, the patient's clinical condition progressively worsened. She experienced increasing pain, development of immobility, and significant exacerbation of bilateral hip symptoms.

Subsequent physical examination revealed external rotation and 2 cm shortening of the left lower extremity. As a result, the patient's pregnancy was terminated surgically by abdominal cesarean section at 36 weeks of gestation. Following delivery, pelvic MRI and plain radiographic examination were performed.

Imaging studies demonstrated a subcapital fracture of the right femoral neck with minimal varus impaction and a subcapital fracture of the left femoral neck with complete displacement. The fractures were classified according to the AO/OTA system as 31-B1.2 on the right and 31-B1.3 on the left femoral neck. According to the Garden classification as type II-III on the right and type IV on the left femoral neck.

Second postoperative day following the cesarean section, surgical treatment was indicated and performed, consisting of bilateral osteosynthesis of the subcapital femoral neck fractures.

### Surgery

The surgery was performed under general anaesthesia. The right femoral neck fracture was addressed first and stabilized using the Femoral Neck System (DePuy Synthes, Zuchwil, Switzerland) without any intraoperative complications. Adequate reduction was achieved, allowing the same fixation technique to be used for the left femoral neck fracture.

Both fractures were reduced on a traction table using traction and internal rotation. Reduction of the left femoral neck fracture was achieved with a shortening of 4 mm, valgus alignment of approximately 10°, and preservation of physiological anteversion on the lateral view. Postoperative radiographs confirmed satisfactory bilateral positioning of the bolt and antirotational screw in both anteroposterior and lateral projections.

### Post-operative period

In the postoperative period, unrestricted range-of-motion exercises of both hip joints were allowed and the pain significantly improved immediately. After one week, the patient was pain-free and able to perform active hip flexion up to 100°. Six weeks after surgery, she began walking with crutch support, having previously mobilized using a wheelchair, and until then she had been able to transfer independently from the bed to the wheelchair without difficulty.

Pharmacological thromboprophylaxis was administered with low-molecular-weight heparin (Fraxiparine, Sanofi, Paris, France) at a dose of 0.6 ml subcutaneously once daily for six weeks postoperatively. Wheelchair-assisted mobilization was recommended to minimise the risk of osteosynthetic failure. The patient was informed about the increased risk of venous thromboembolism as well as the practical limitations related to newborn care.

Six months after surgery, the patient developed progressive pain in the left hip, accompanied by approximately 1-cm shortening of the lower extremity and deterioration of functional status. Follow-up radiographs revealed avascular necrosis of the weight-bearing portion of the left femoral head. Based on these findings, the previously implanted osteosynthetic material was removed, and a cementless total hip arthroplasty was subsequently performed.

**Fig. 1:**
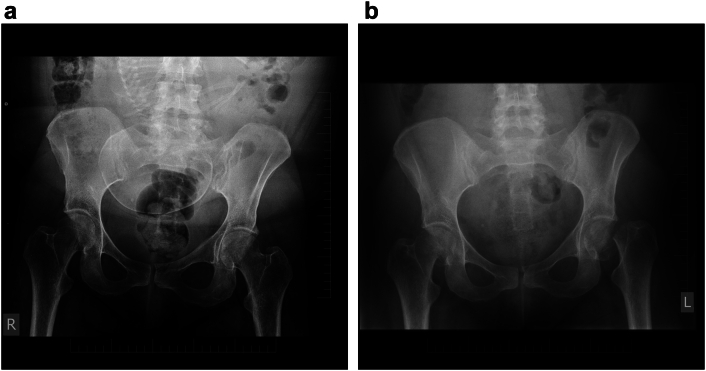
Pelvic radiograph of a pregnant patient with transient osteoporosis of the hip and fractures as a complication of treatment. 1a, - Radiograph demonstrating no pathological findings on initial imaging at admission. 1b, - Radiograph demonstrating bilateral subcapital femoral neck fractures identified postpartum.

**Fig. 2:**
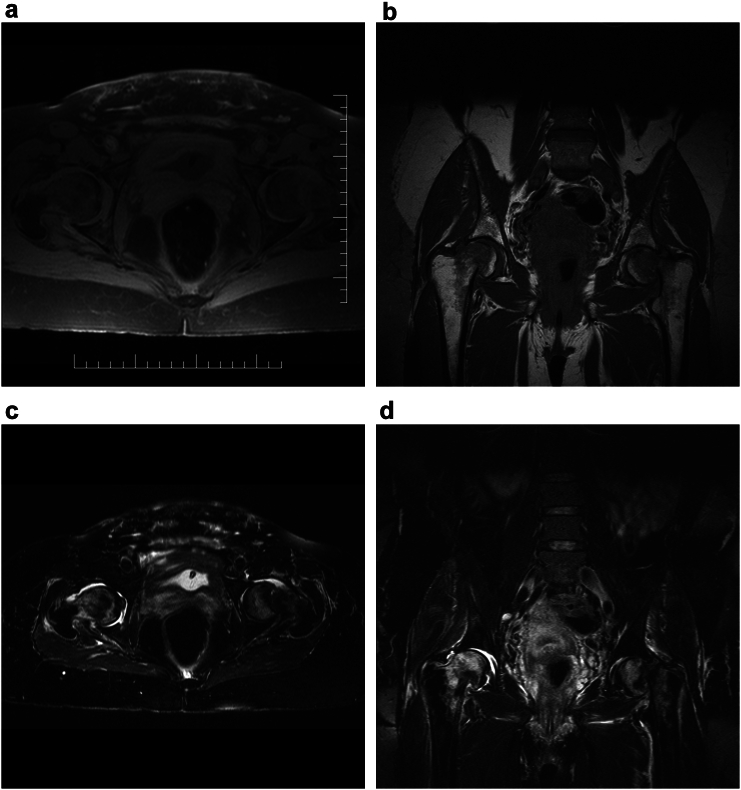
MRI of the affected hip joints in a 46-year-old woman performed 1 day postpartum. 2a, - Reduced signal intensity on T1-weighted transverse images. 2b, - Reduced signal intensity on T1-weighted coronal images. 2c, - Increased signal intensity on T2-weighted transverse images. 2d, - Increased signal intensity on T2-weighted coronal images.

**Fig. 3:**
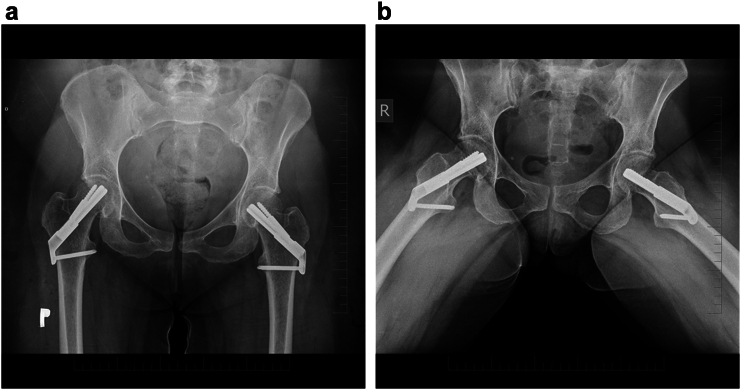
Radiograph of the affected hips 1 day postoperatively, after closed reduction and fixation with the Femoral Neck System (FNS, DePuy Synthes). 3a, - Anteroposterior (AP) view of the pelvis. 3b, - Axial view of the pelvis.

**Fig. 4:**
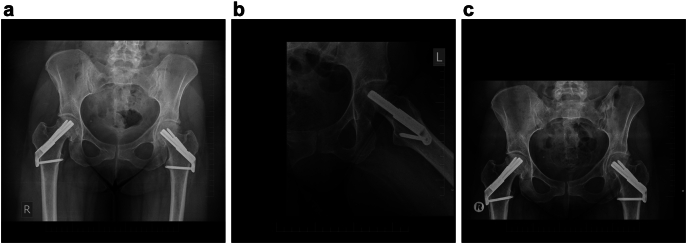
Radiographs of the affected hips at 3 and 6 months postoperatively. 4a., - Radiograph after bilateral femoral neck osteosynthesis, 3 months postoperatively- (AP) view. 4b, - Axial view 6 months postoperatively demonstrating partial avascular necrosis of the left femoral head. 4c, - Anteroposterior view 6 months postoperatively demonstrating partial avascular necrosis of the left femoral head.

**Fig. 5:**
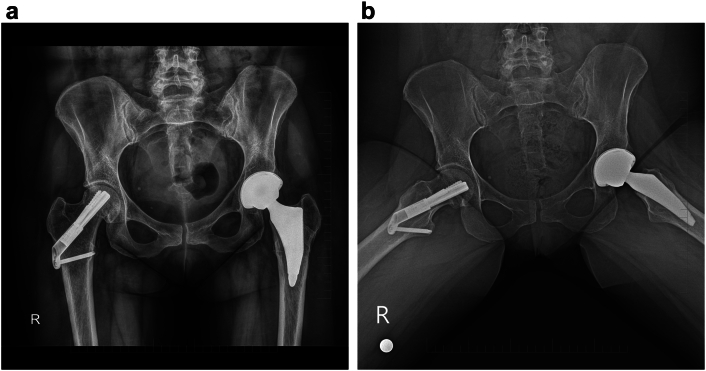
Radiographs 4 years after total hip replacement performed for avascular necrosis of the femoral head. 5a, - Anteroposterior (AP) view of the pelvis. 5b, - Axial view of the pelvis.

Four years after surgery, the patient was able to bear full weight without restrictions in her routine daily activities. She reported a good functional outcome with pain-free range of motion in both hips. Active range of motion (AROM) of both hip joints was as follows: S 10–0–120°, F 40–0–20°, T 20–0–20° (Fig. 1, Fig. 2, Fig. 3, Fig. 4, Fig. 5).

## Discussion

Transient osteoporosis of the hip (TOH) is a rare, reversible form of bone marrow oedema syndrome (BMES) but remians a controversial condition. Clinically, it presents with progressive hip pain, marked limitation of mobility and reversible osteopenia of the proximal femur. Due to its low incidence and nonspecific clinical features, the condition may be misdiagnosed, increasing the risk of serious complications, particularly pathological fractures or avascular necrosis. These complications are more frequently reported in pregnant patients [1]. A characteristic feature of transient osteoporosis of the hip (TOHP) in pregnancy is spontaneous regression of symptoms and radiological findings within months postpartum [1].

TOH occurs more commonly in middle-aged men, but pregnancy is an additional risk factor. Severe forms of TOHP typically manifest in the third trimester or the immediate postpartum period. Bilateral involvement during pregnancy, as observed in this case, has been reported in previous studies [2], [3]. According to the literature, approximately 25% of symptomatic TOH cases in pregnancy are bilateral [4].

The etiology of transient osteoporosis of the hip in pregnancy remains unclear. Several hypotheses have been proposed including vascular factors such as impaired venous outflow, bone marrow oedema, and increased pressure [5]. These perfusion changes can result in ischemia [6]. Other theories include neurovascular dysfunction, complex regional pain syndrome, hormonal changes, and mechanical or metabolic stress [7], [8], [9]. Literature describes TOHP as a multifactorial condition with several associated risk factors [10]. In this case, advanced maternal age (primigravida vetus), obesity and pregnancy achieved via in vitro fertilization are considered potential risk factors. The patient's history of distal radius and distal humerus fractures may indicate underlying osteopenia. Hypothyroidism has also been reported as a contributing risk factor [11]. In a retrospective study of 34 women with TOHP, common risk factors included advanced maternal age, lower BMI, positive family history of osteoporosis, and pregnancies achieved via assisted reproduction, including IVF [4]. Other reported risk factors include reduced physical activity, or significant childhood dental problems [10].

Prevention of pathological fractures in TOHP requires clinical vigilance and close collaboration between obstetricians and orthopaedic surgeons. Awareness of clinical features, timely diagnosis, optimized management strategies, and prevention of complications such as fractures or progression to avascular necrosis are key aspects of patient care.

Plain radiography is usually unremarkable in the early stages unless fractures, osteonecrosis, or other concomitant pathology are present. One to two months symptoms onset, radiographs may reveal osteopenia of the femoral head and neck, as well as changes in the trochanteric region. Magnetic resonance imaging (MRI) is currently the standard method for early diagnosis of TOH in pregnancy, particularly in patients with progressive, severe hip pain. MRI is considered low-risk during pregnancy and provides high sensitivity for differentiating TOH from other hip pathologies like fractures, degenerative processes, inflammatory diseases or neoplasia. It allows detection of bone marrow oedema in the femoral head and neck, fracture lines, signs of necrosis, and joint effusion. MRI typically demonstrates low signal intensity on T1-weighted sequences and high signal intensity on T2-weighted sequences. In the presence of a fracture, a thin low-signal band is visible within the edematous region, with surrounding changes consistent with bone marrow edema [8].

Data on the incidence of fractures in TOHP are limited. Subcapital femoral neck fractures are a rare but serious complication most commonly occurring during first pregnancy [12]. Available literature suggests that insufficient fractures may occur in approximately 10–25% of TOHP cases [1], [10], though exact incidence remains unclear due to the small number of published cases. Bilateral femoral neck fractures account for approximately one-third of cases, while unilateral involvement is observed in the remaining two-thirds.

In cases of fracture, surgical intervention may be postponed until the postpartum period in selected situations, although pregnancy itself is not a contraindication for surgery [13]. Bone healing potential remains preserved despite osteopenia and conservative approach with osteosynthesis is a viable option. Despite these findings, therapeutic options include closed reduction with screw fixation, dynamic hip screw (DHS) fixation, or total hip arthroplasty, each with distinct advantages. Vergara-Ferrer et al. reported 16 hip fracture cases managed with various surgical options, in all but one case, fractures treated with osteosynthesis healed without subsequent necrosis, regardless of time to diagnosis [14]. Yassin et al. described the development of avascular necrosis in a case of bilateral subcapital fracture associated with TOHP, affecting the left hip, which was classified as Garden type III–IV and treated with closed reduction and internal fixation using screws [15]. Willis-Owen described a similar case with identical fracture types according to the Garden classification as in our patient. The fractures were managed with closed reduction and dynamic hip screw (DHS) fixation. The fractures healed without avascular necrosis or implant failure, however, the follow-up period was short, limited to six months [16].

Due to limited data, there is no consensus on the optimal type of osteosynthesis in this patient population. The use of the Femoral Neck System (FNS) as a relatively new implant and its specific application in subcapital fractures associated with TOHP has likely not been previously reported. FNS provides greater stability compared to cannulated screws and comparable stability to DHS [17]. Based on the meta-analysis, the use of the FNS is associated with a lower risk of avascular necrosis than dynamic hip screws or cannulated screws [18]. In our case, one fracture was stable and undisplaced, while the other was displaced. After reduction on the traction table, both fractures were classified as Pauwels type I–II. Bilateral osteosynthesis was performed after thorough patient counselling and consent, with indication based on achievable closed reduction of cortical lines, preservation of anatomical anteversion, and absence of varus deformity.

During follow-up, the fractures healed without secondary displacement. However, the patient later developed symptomatic avascular necrosis with a subchondral fracture of the dorsal part of the femoral head. Removal of the FNS implant and subsequent cementless total hip arthroplasty resulted in a good functional and radiological outcome at mid-term follow-up.

## CRediT authorship contribution statement

**Zoltan Cibula:** Writing – original draft, Methodology, Conceptualization. **Jozef Cabala:** Project administration, Methodology, Investigation. **Milan Cipkala:** Writing – original draft, Visualization. **Peter Lisý:** Project administration, Methodology, Data curation, Conceptualization. **Juraj Cabala:** Writing – review & editing, Software.

## Informed consent

Informed consent was obtained from the participant, and all investigations were conducted in conformity with ethical principles.

## IRB approval

All study participants provided informed consent, and the study design was approved by an ethics review board.

## Human and animal rights disclosure

All institution involved in this work have approved the human protocol for this investigation.

## Declaration of competing interest

The authors state that there are no conflicts of interest regarding the publication of this article.
